# NXC736, a Functional Antagonist of S1P_4_, Attenuates Brain Injury in Mice with Permanent Ischemic Stroke

**DOI:** 10.3390/molecules30234504

**Published:** 2025-11-21

**Authors:** Nikita Basnet, Supriya Tiwari, Kyung Hee Choi, Donghee Kim, Ji Woong Choi

**Affiliations:** College of Pharmacy and Gachon Institute of Pharmaceutical Sciences, Gachon University, 191 Hambakmoero, Yeonsu-gu, Incheon 21936, Republic of Korea

**Keywords:** NXC736, S1P_4_, permanent ischemic stroke, permanent middle cerebral artery occlusion (pMCAO)

## Abstract

Stroke is the leading cause of death and long-term disability worldwide, with ischemic stroke accounting for nearly 87% of all cases. Vascular occlusion, a key pathological event in ischemic stroke, has been reliably reproduced in preclinical studies using permanent ischemic stroke models. This study demonstrated the neuroprotective effect of NXC736, a functional antagonist of sphingosine-1-phosphate receptor 4 (S1P_4_, currently in phase II clinical trials for alopecia areata), against acute injury in mice with permanent middle cerebral artery occlusion (pMCAO). pMCAO-challenged mice received oral NXC736 1 h after occlusion. NXC736 demonstrated substantial therapeutic activity against permanent ischemic stroke by attenuating pMCAO-induced acute brain infarction, neurological deficits, and apoptosis. Additionally, NXC736 reduced blood–brain barrier disruption and edema in the injured brain. Moreover, NXC736 reduced microglial activation and proliferation, oxidative stress, and suppressed pro-inflammatory cytokine expression, suggesting that the efficacy of NXC736 in permanent ischemic stroke is associated with the suppression of neuroinflammatory responses. Mechanistically, we found that NXC736-mediated neuroprotective effects were dependent on the inactivation of NF-κB and MAPKs, including ERK1/2, JNK, and p38. Collectively, our findings indicate that NXC736 is an effective neuroprotective drug for permanent ischemic brain stroke, highlighting S1P_4_ as a promising therapeutic target for ischemic stroke.

## 1. Introduction

Sphingosine 1-phosphate (S1P) is a pivotal bioactive lipid that signals through five G protein-coupled receptors (S1P_1–5_) to regulate multiple physiological and pathological functions, including vascular integrity, immune cell trafficking, and neuroinflammatory responses [1]. Receptor-mediated S1P signaling is involved in the pathogenesis of various autoimmune and neuroinflammatory diseases. Notably, S1P receptors are molecular targets for drug development [2]. For example, FTY720 (fingolimod), which binds to four receptors (S1P_1,3,4, and 5_), is used as a clinically to treat multiple sclerosis [3,4] and has been investigated in ischemic stroke [5,6], neurodegenerative diseases [7,8], cancers [9,10], inflammatory bowel disease [11], and organ transplantation [12].

NXC736 (also known as SLB736) is a recently developed FTY720 analog [13,14]. It has been shown to act selectively on S1P4 based on β-arrestin recruitment assays, in which NXC736 exhibits high efficacy solely at S1P_4_ [13,14]. Furthermore, NXC736 has been revealed as a functional antagonist against S1P_4_ by blocking the receptor recycling to the cell surface, thereby removing S1P_4_ from the cell membrane [14]. A phase II clinical trial evaluating oral NXC736 for alopecia areata is currently ongoing (ClinicalTrials.gov Identifier: NCT06104839). Moreover, the potential of NXC736 has been examined in lung fibrosis [15] and as a pharmacological tool to elucidate the role of S1P_4_ in metabolic dysfunction-associated steatohepatitis (MASH) [14] and transient ischemic stroke [13]. We have previously identified a crucial pathogenic role of S1P_4_ in transient ischemic stroke using pharmacological (NXC736) and genetic approaches [13]. NXC736 exerts its neuroprotective effects against transient ischemic stroke by reducing brain damage and mitigating pathogenic events such as microglial activation and proliferation, as well as NLRP3 inflammasome activation [13].

However, the potential effect of NXC736 on permanent ischemic stroke remains unknown. Permanent ischemic stroke is associated with exacerbation of neuroinflammation, extensive leukocyte infiltration, and larger infarction volumes, indicating a more severe pathological state [16,17]. Therefore, NXC736 may exert therapeutic effects against permanent ischemic stroke. Moreover, considering the remarkable neuroprotective effects mediated by NXC736 in a transient ischemic stroke model, its efficacy in treating permanent ischemia warrants further investigation. Importantly, the permanent ischemic model more closely mimics the acute phase of the majority of clinical stroke cases compared with other middle cerebral artery occlusion (MCAO) models [18], making it a clinically relevant model to evaluate the therapeutic potential of NXC736 in stroke without reperfusion. This study aimed to investigate the neuroprotective effects of NXC736, a functional antagonist of S1P_4_, and to elucidate its underlying mechanisms in a mouse model of permanent ischemic stroke.

## 2. Results

### 2.1. NXC736 Attenuates Brain Damage and Improves Neurological Functions One and Three Days After pMCAO Challenge

**Figure 1:**
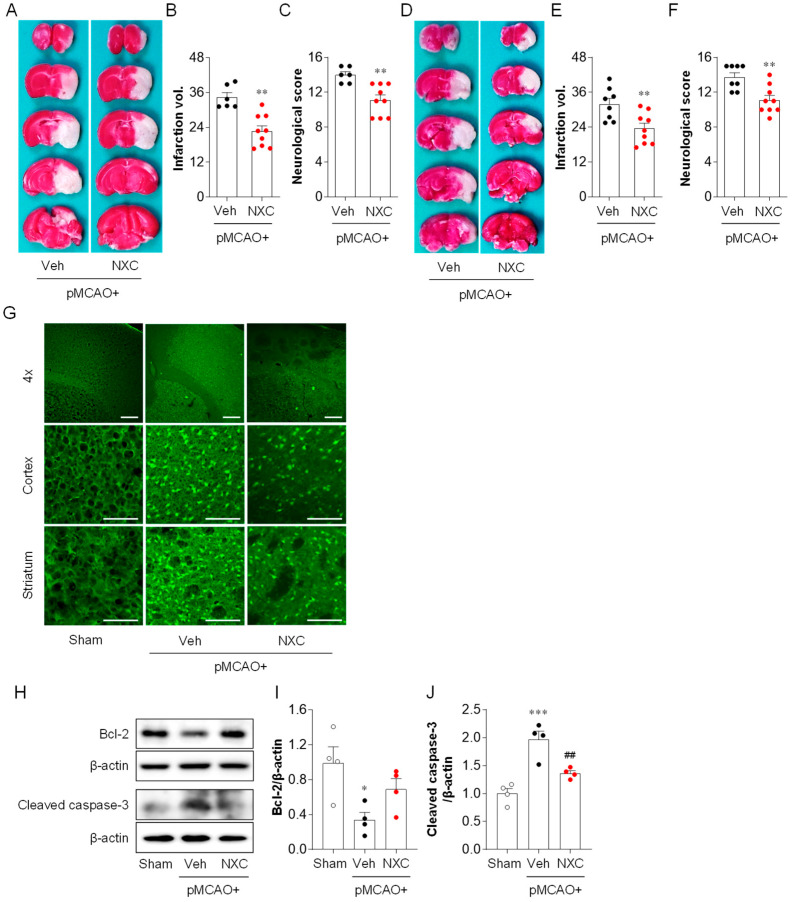
NXC736 administration attenuates brain damage in pMCAO-challenged mice. Mice underwent pMCAO or sham surgery, and brains were harvested at one or three days post-surgery. Vehicle (Veh, 10% Tween 80) or NXC736 (NXC, 3 mg/kg, *p.o.*) was administered 1 h post-occlusion. (**A**–**F**) Effect of NXC736 on brain injury was determined one or three days later. The effects of NXC736 on brain infarction volume and neurological score were analyzed. Infarction volume and neurological scores were determined at one (**A**–**C**) and three (**D**–**F**) days after pMCAO. Representative images of TTC-stained brain sections (**A**,**D**), quantification of infarction volume (**B**,**E**), and neurological score (**C**,**F**) are shown. *n* = 6–9 mice per group. ** *p* < 0.01 vs. vehicle-administered pMCAO group. Mann–Whitney test for (**B**,**C**) and (**F**); Student’s *t*-test for (**E**). (**G**–**J**) Effects of NXC736 on cell death were analyzed one day after pMCAO. (**G**) Fluoro-Jade B (FJB) staining was performed to detect brain cell death in the cortex and striatum regions. Scale bars, 200 µm (**top**) and 50 µm (**middle** and **bottom**). (**H**–**J**) Expression of apoptosis-related proteins Bcl-2 and cleaved caspase-3 was examined by Western blot analysis. Representative immunoblots (**H**) and quantification (**I**,**J**) are shown. *n* = 4 mice per group. * *p* < 0.05 or *** *p* < 0.001 vs. sham; ^##^ *p* < 0.01 vs. vehicle-administered pMCAO group. Tukey’s post hoc test for (**I**,**J**).

Brain damage was determined by evaluating brain infarct volume and neurological deficit score one and three days after pMCAO. Mice treated with vehicle developed severe infarction in the damaged hemisphere, with a larger infarction volume observed at both one (Figure 1A,B) and three days post-ischemia (Figure 1D,E). In contrast, NXC736-treated mice demonstrated a significantly smaller brain infarct volume at both time points compared with that in the vehicle-treated group (Figure 1A,B,D,E). Moreover, the modified neurological score was remarkably improved in the NXC736-treated group compared with that in the vehicle-treated group at both one (Figure 1C) and three days (Figure 1F). To confirm the neuroprotective effects of NXC736, Fluoro-Jade B (FJB) staining was performed to assess brain cell death (Figure 1G). NXC736 treatment significantly reduced the number of FJB-positive degenerating neurons compared with vehicle treatment (Figure 1G). To determine whether the NXC736-mediated neuroprotective effects were associated with the attenuation of apoptosis in ischemic stroke, we examined Bcl-2, an apoptosis-regulating protein, and cleaved-caspase-3, a key marker of apoptosis, using Western blot analysis (Figure 1H–J). Administration of NXC736 significantly upregulated the expression of anti-apoptotic protein Bcl-2 (Figure 1H,I) and notably downregulated cleaved caspase-3 expression (Figure 1H,J). These results indicate that NXC736 effectively reduces brain damage, improves neurological functional outcomes, and suppresses apoptosis in pMCAO-challenged mice.

### 2.2. NXC736 Ameliorates BBB Disruption and Reduces Brain Edema in the Post-Ischemic Brain After pMCAO Challenge

**Figure 2:**
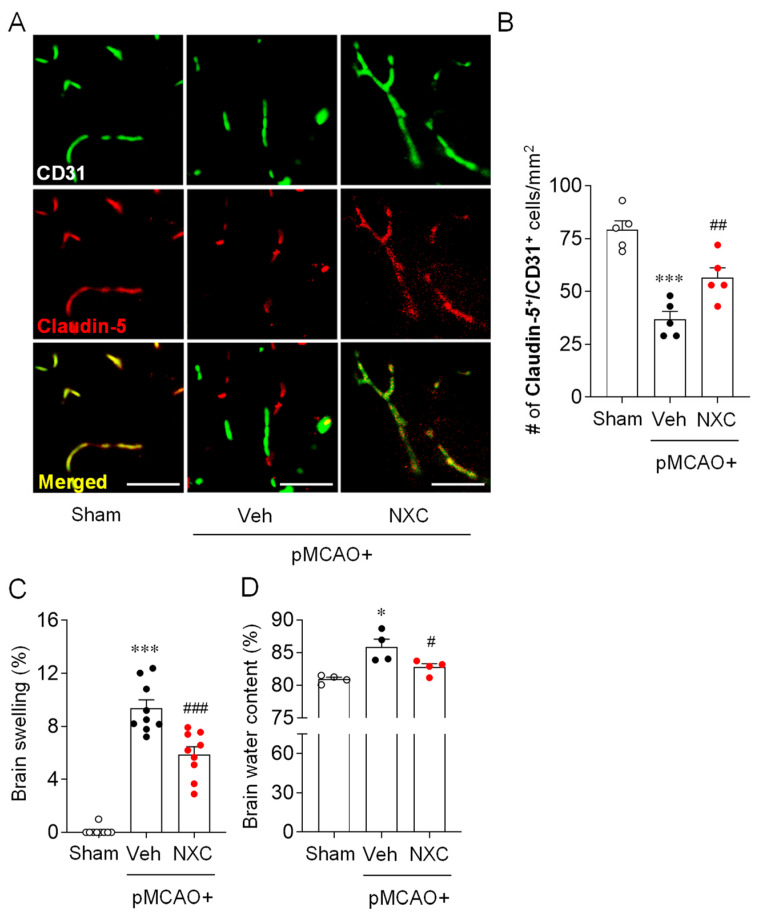
NXC736 administration attenuates BBB disruption and reduces brain edema in pMCAO-challenged mice. Mice underwent pMCAO or sham surgery, and brains were harvested one day post-surgery. Vehicle (Veh, 10% Tween 80) or NXC736 (NXC, 3 mg/kg, *p.o.*) was administered 1 h post-occlusion. (**A**,**B**) Effects of NXC736 on BBB disruption were determined by double immunofluorescence for Claudin-5 (red) and CD31 (green) in ischemic core regions one day post-pMCAO. Representative images of Claudin-5^+^CD31^+^ cells (**A**) and quantification of the number of Claudin-5^+^CD31^+^ cells (**B**) are shown. Scale bars, 50 µm. Tukey’s post hoc test for (**B**–**D**) Effects of NXC736 on brain edema were evaluated by measuring brain swelling using ImageJ (Kaplan method) (**C**) and brain water content by the dry-wet method (**D**). *n* = 4–5 mice per group. * *p* < 0.05 or *** *p* < 0.001 vs. sham; ^#^ *p* < 0.05, ^##^ *p* < 0.01, or ^###^ *p* < 0.001 vs. vehicle-administered pMCAO group. Dunn’s post hoc test for (**C**,**D**).

Stroke-induced BBB disruption facilitates the ingress of cytotoxic substances, promotes vasogenic brain edema, and may lead to hemorrhagic transformation [19]. To determine whether NXC736 mitigates BBB disruption in the permanently ischemic brain, we performed Claudin-5/CD31 double immunofluorescence analysis (Figure 2A,B). BBB disruption was markedly increased in the vehicle-treated group, and treatment with NXC736 significantly preserved BBB integrity, as indicated by improved Claudin-5/CD31 colocalization (Figure 2A,B). Furthermore, we assessed brain edema by evaluating brain swelling and water content (Figure 2C,D). Compared with the vehicle-treated group, the NXC736-treated group exhibited a significant reduction in brain swelling (Figure 2C). Similarly, the elevated brain water content observed in vehicle-treated mice was markedly reduced following NXC736 administration (Figure 2D). These results suggest that NXC736 not only improves BBB dysfunction but also effectively reduces brain edema in the post-ischemic brains of pMCAO-challenged mice.

### 2.3. NXC736 Attenuates Microglial Activation and Proliferation in Post-Ischemic Brain After pMCAO Challenge

**Figure 3:**
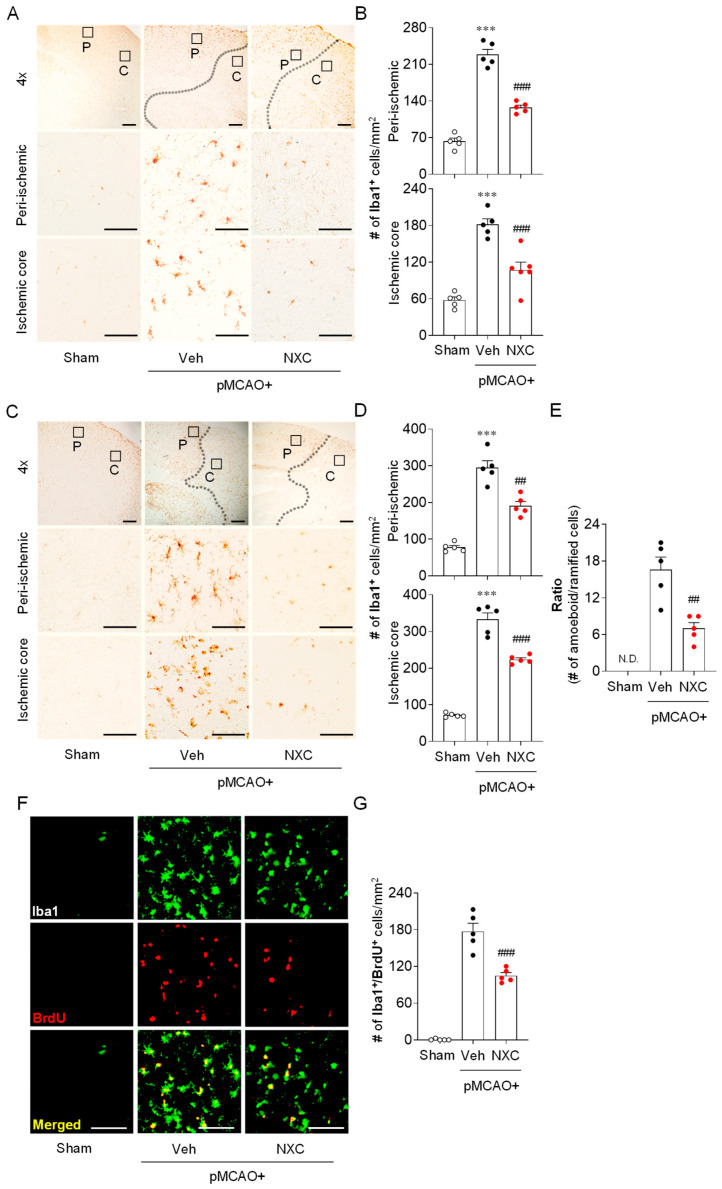
NXC736 administration attenuates microglial activation and proliferation in the injured brain of pMCAO-challenged mice. Mice underwent pMCAO or sham surgery, and brains were harvested one or three days post-surgery. Vehicle (Veh, 10% Tween 80) or NXC736 (NXC, 3 mg/kg, *p.o.*) was administered 1 h post-occlusion. (**A**–**E**) Effects of NXC736 on microglial activation in peri-ischemic (“P”) and ischemic core (“C”) regions were determined one (**A**,**B**) and three (**C**–**E**) days after pMCAO challenge by performing Iba1 immunohistochemistry. Representative images of Iba1^+^ cells (**A**,**C**) and quantification of the number of Iba1^+^ cells in each region (**B**,**D**) are shown. Scale bars, 200 µm (top panels in (**A**,**C**)) and 50 µm (middle and bottom panels in (**A**,**C**). (**E**) Quantification of the ratio of amoeboid-to-ramified microglia in the ischemic core regions three days after pMCAO challenge. In the upper panel, the diagram box depicts the cerebral regions with magnified views in the middle and bottom panels. Dotted lines demarcate peri-ischemic and core regions. Student’s *t*-test for (**E**). (**F**,**G**) Effects of NXC736 on microglial proliferation in the penumbra region were analyzed by double immunofluorescence of Iba1 (green) and BrdU (red). Representative images of BrdU^+^Iba1^+^ cells (**F**) and quantification of the number of Iba1^+^BrdU^+^ cells (**G**) are shown. Scale bars, 50 µm. N.D., not detected. *n* = 5 mice per group. *** *p* < 0.001 vs. sham; ^##^ *p* < 0.01 or ^###^ *p* < 0.001 vs. vehicle-treated post-ischemic brain after pMCAO challenge. Tukey’s post hoc test for (**B**,**D**); Student’s *t*-test for (**E**,**G**).

To investigate whether NXC736 attenuates microglial activation in post-ischemic brains, Iba1 immunohistochemistry was performed in the peri-ischemic and ischemic core regions one and three days after pMCAO. In the vehicle-treated pMCAO group, the number of Iba1^+^ cells was notably higher in both the peri-ischemic and ischemic core regions at one (Figure 3A,B) and three days (Figure 3C,D) compared with the sham group. The observed increase was significantly attenuated in the NXC736-treated group (Figure 3A–D). Notably, NXC736 treatment also significantly reduced the number of amoeboid microglia, as evidenced by the decreased amoeboid-to-ramified ratio in injured brains three days after the pMCAO challenge (Figure 3C,E). Microglial proliferation, a common response in the injured brain, typically peaks between 3 and 5 days after an ischemic stroke challenge [20]. To determine whether NXC736 affects microglial proliferation, we performed histological analysis using Iba1/bromodeoxyuridine (BrdU) double immunofluorescence in the penumbral region of the post-pMCAO brains. The number of Iba1^+^BrdU^+^ cells was significantly increased in the vehicle-treated pMCAO group (Figure 3F,G), indicating robust microglial proliferation. Conversely, NXC736 treatment significantly decreased the number of proliferating microglia in the pMCAO-challenged brain (Figure 3F,G). These results indicate that the S1P_4_ antagonist NXC736 effectively suppresses both microglial activation and proliferation in the post-ischemic brain following pMCAO.

### 2.4. NXC736 Attenuates Oxidative Stress in the Post-Ischemic Brain After pMCAO Challenge

**Figure 4:**
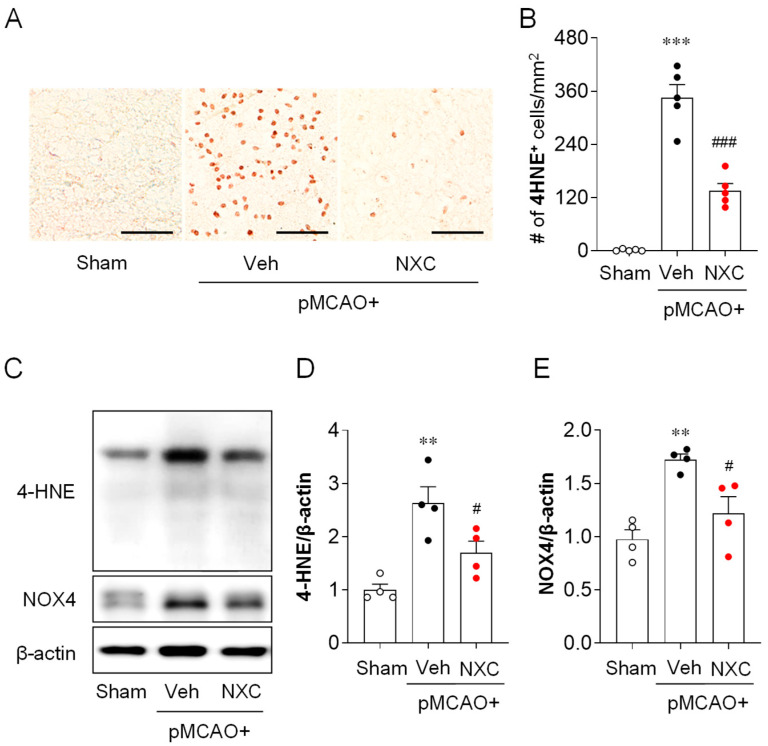
NXC736 administration attenuates oxidative stress in the injured brain of pMCAO-challenged mice. Mice underwent pMCAO or sham surgery, and brains were harvested one day after surgery. Vehicle (Veh; 10% Tween 80) or NXC736 (NXC; 3 mg/kg, *p.o.*) was administered 1 h post-occlusion. (**A**,**B**) Effects of NXC736 on oxidative stress were assessed by immunohistochemical analysis of 4-hydroxynonenal (4-HNE) in the ischemic core region. Brown staining indicates 4-HNE–positive cells. Representative images (**A**) and quantification (**B**) of 4-HNE–positive cells are shown. Scale bar = 50 μm. *n* = 5 mice per group. *** *p* < 0.001 vs. sham; *^###^ p* < 0.001 vs. vehicle-treated group. Tukey’s post hoc test for (**B**). (**C**–**E**) Western blot analysis showing the expression of 4-HNE and NADPH oxidase 4 (NOX4) in the post-ischemic brain. Representative immunoblots (**C**) and quantification (**D**,**E**) are shown. β-actin served as the loading control. *n* = 5 mice per group. ** *p* < 0.01 vs. sham; ^#^ *p* < 0.05 vs. vehicle-treated group. Tukey’s post hoc test for (**D**,**E**).

To evaluate whether NXC736 mitigates oxidative stress after pMCAO, we examined the expression of 4-hydroxynonenal (4-HNE), a marker of lipid peroxidation, and NADPH oxidase 4 (NOX4) in the damaged hemisphere one day after occlusion. The number of 4-HNE-positive cells in the ischemic core region was markedly higher in the vehicle-treated pMCAO group than in the sham group (Figure 4A,B). This elevation was significantly reduced in the NXC736-treated group. Consistent with the immunohistochemical findings, the protein levels of 4-HNE and NOX4 were higher in the vehicle-treated pMCAO group (Figure 4C–E). However, NXC736 treatment significantly reduced the expression levels of both 4-HNE and NOX4 compared with the vehicle group. These results indicate that NXC736 effectively suppresses oxidative stress in the post-ischemic brain following pMCAO.

### 2.5. NXC736 Downregulates Pro-Inflammatory Cytokine mRNA Expression Without Affecting Anti-Inflammatory Cytokine Expression in Post-Ischemic Brain After pMCAO Challenge

**Figure 5:**
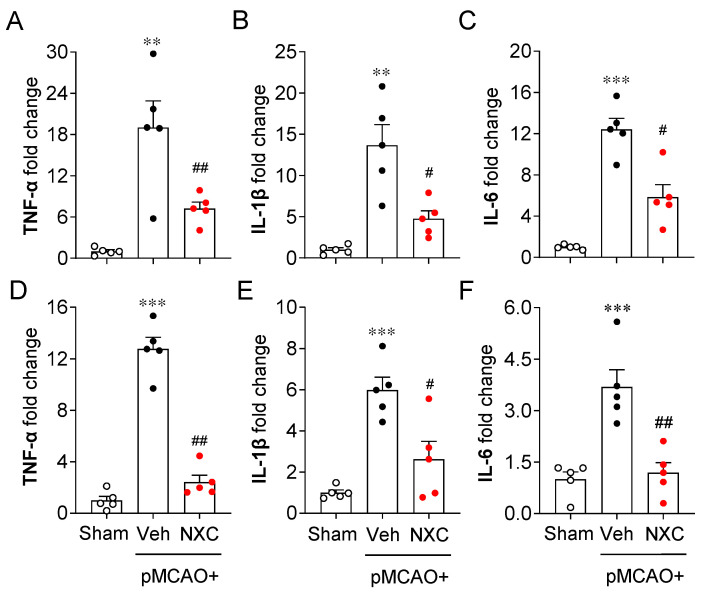
NXC736 administration downregulates pro-inflammatory cytokine expression in injured brains of pMCAO-challenged mice. Mice underwent pMCAO or sham surgery, and brains were harvested one or three days post-surgery. Vehicle (Veh, 10% Tween 80) or NXC736 (NXC, 3 mg/kg, *p.o.*) was administered 1 h post-occlusion. Effects of NXC736 on mRNA expression of pro-inflammatory cytokines (TNF-α, IL-1β, and IL-6) were analyzed in the damaged hemisphere of the post-pMCAO brain at one (**A**–**C**) and three (**D**–**F**) days by RT-qPCR analysis. Changes in the mRNA expression levels of TNF-α (**A**,**D**), IL-1β (**B**,**E**), and IL-6 (**C**,**F**) are shown. *n* = 5 mice per group. ** *p* < 0.01 or *** *p* < 0.001 vs. sham; ^#^ *p* < 0.05 or ^##^ *p* < 0.01 vs. vehicle-treated post-ischemic brain after pMCAO challenge. Tukey’s post hoc test for (**A**–**C**,**E**,**F**); Dunn’s post hoc test for (**D**).

**Figure 6:**
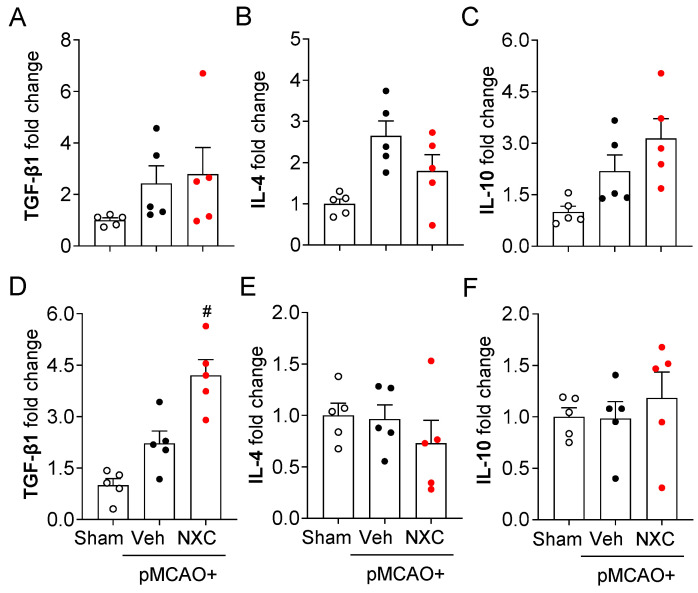
NXC736 administration does not alter the expression of anti-inflammatory cytokines in the injured brain of pMCAO-challenged mice. Mice underwent pMCAO or sham surgery, and brains were harvested one or three days post-surgery. Vehicle (Veh, 10% Tween 80) or NXC736 (NXC, 3 mg/kg, *p.o.*) was administered 1 h post-occlusion. Effects of NXC736 on mRNA expression of anti-inflammatory cytokines (TGF-β1, IL-4, and IL-10) were determined in the damaged hemisphere of the post-pMCAO brain at one (**A**–**C**) and three (**D**–**F**) days by RT-qPCR analysis. Changes in the mRNA expression levels of TGF-β1 (**A**,**D**), IL-10 (**B**,**E**), and IL-4 (**C**,**F**) are shown. *n* = 5 mice per group. ^#^ *p* < 0.05 vs. vehicle-treated post-ischemic brain after pMCAO challenge. Tukey’s post hoc test for (**D**).

The production and release of pro- and anti-inflammatory cytokines constitute a key pathogenic event in cerebral ischemia. Activated microglia trigger the production of inflammatory cytokines in the ischemic brain [21,22]. To determine whether NXC736 influences pro- and anti-inflammatory responses in the permanently ischemic brain, we detected the mRNA expression of both pro- and anti-inflammatory cytokines in the damaged region at one and three days post-ischemia (Figure 5 and Figure 6). In the vehicle-treated group, expression levels of pro-inflammatory cytokines (tumor necrosis factor [TNF]-α, interleukin [IL]-1β, and IL-6) were significantly elevated in the post-ischemic brain (Figure 5A–F). In contrast, NXC736 treatment markedly downregulated the expression of these pro-inflammatory cytokines (Figure 5A–F). Administration of NXC736 did not alter mRNA expression levels of anti-inflammatory cytokines, including IL-4 and IL-10, in the brains of pMCAO-challenged mice (Figure 6A–C,E,F). Interestingly, NXC736 treatment significantly upregulated the mRNA expression of transforming growth factor (TGF)-β1, another anti-inflammatory cytokine, by two-fold at three days post-ischemia (Figure 6D). These findings suggest that NXC736 selectively modulates the inflammatory response in the post-ischemic brain by suppressing pro-inflammatory cytokines while sparing or enhancing certain anti-inflammatory mediators, such as TGF-β1.

### 2.6. NXC736 Attenuates NF-κB Upregulation in Activated Microglia and MAPK Activation in the Post-Ischemic Brain After pMCAO Challenge

**Figure 7:**
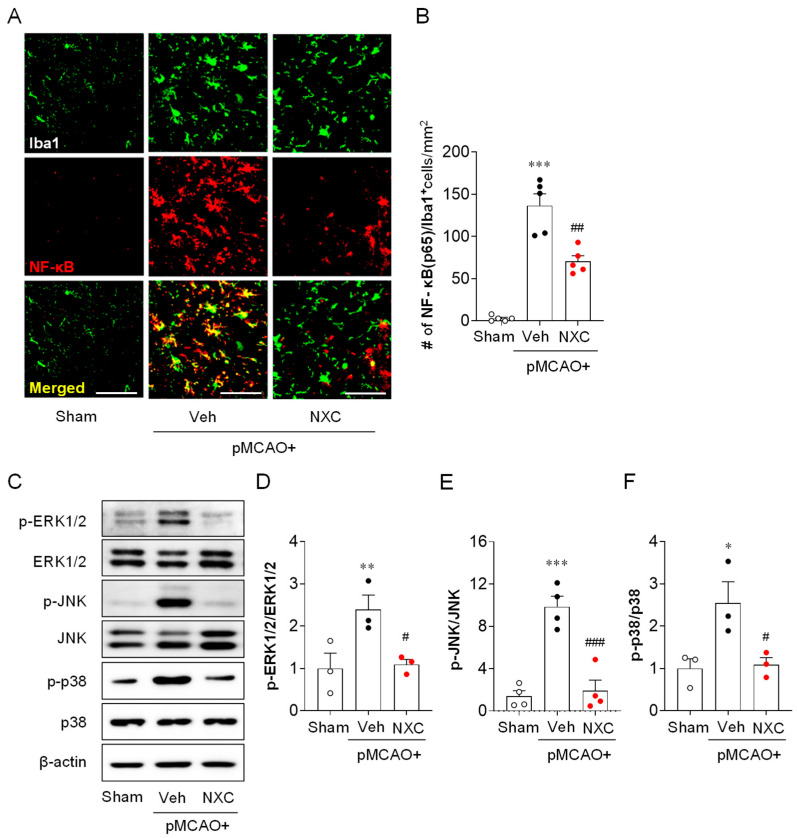
NXC736 administration attenuates NF-κB upregulation and MAPK activation in injured brains of pMCAO-challenged mice. Mice underwent pMCAO or sham surgery, and brains were harvested one day post-surgery. Vehicle (Veh, 10% Tween 80) or NXC736 (NXC, 3 mg/kg, *p.o.*) was administered 1 h post-occlusion. (**A**,**B**) Effects of NXC736 on NF-κB expression in the injured brain were determined by NF-κB/Iba1 double immunofluorescence analysis at one day after pMCAO. Representative images of NF-κB^+^Iba1^+^ cells (**A**) and quantification of the number of NF-κB^+^Iba1^+^ cells (**B**) are shown. Scale bar, 50 µm. *n* = 5 mice per group. *** *p* < 0.001 vs. sham; ^##^ *p* < 0.01 vs. vehicle-administered pMCAO group. Tukey’s post hoc test for (**B**). (**C**–**F**) Effects of NXC736 on MAPK activation in damaged brains were assessed by Western blot analysis one day after pMCAO. Representative Western blots of phosphorylated MAPKs (p-ERK1/2, p-JNK, and p-p38), total MAPKs (ERK1/2, JNK, and p38), and β-actin (**C**) are shown. β-actin served as the loading control. Quantification of activation of ERK1/2 (**D**), JNK (**E**), and p38 (**F**) is shown. *n* = 3 mice per group. * *p* < 0.05 or ** *p* < 0.01 vs. sham; ^#^ *p* < 0.05 or ^###^ *p* < 0.001 vs. vehicle-treated post-ischemic brain after pMCAO challenge. Tukey’s post hoc test for (**D**–**F**).

In ischemic stroke, NF-κB and MAPK signaling pathways contribute to brain injury [23]. To determine whether NXC736 influences NF-κB expression in activated microglia, we performed NF-κB/Iba1 double immunofluorescence analysis (Figure 7A,B). In the one-day post-ischemic brain, NF-κB expression in Iba1-positive microglia was markedly increased in the vehicle-treated group (Figure 7A,B). Conversely, NXC736 treatment significantly reduced microglial NF-κB expression at this time point (Figure 6A,B), suggesting that NXC736 suppresses NF-κB activation in activated microglia following pMCAO. To examine the effect of NXC736 on the MAPK pathway, a well-known effector pathway for pro-inflammatory cytokines [13], we assessed MAPK activation by measuring the phosphorylation of ERK1/2, p38, and JNK in the damaged hemisphere one day after pMCAO (Figure 7C–F). Phosphorylation of all MAPKs was significantly elevated in the vehicle-treated group (Figure 7C–F), while administration of NXC736 effectively attenuated the phosphorylation of ERK1/2, p38, and JNK (Figure 7C–F). These data indicate that NXC736 effectively modulates key inflammatory signaling pathways by suppressing NF-κB upregulation in activated microglia and MAPK activation in the post-ischemic brain after the pMCAO challenge.

## 3. Discussion

In this study, we demonstrated that the S1P_4_ functional antagonist, NXC736, exerts neuroprotective effects against permanent ischemic stroke. NXC736 significantly attenuated brain injury in the acute phase, i.e., within one or three days post-ischemia, indicating its efficacy in alleviating early brain damage in pMCAO-challenged mice. Notably, this study demonstrates that NXC736 can modulate several pathogenic events in permanent ischemic stroke: (i) attenuates brain injury by improving neurological scores, reducing infarction volume, and cellular apoptosis; (ii) ameliorates BBB dysfunction and reduces brain edema; (iii) suppresses microglial activation and proliferation; (iv) attenuates upregulation of pro-inflammatory cytokine expression in injured brains; and (v) inhibits upregulation of NF-κB and activation of MAPK signaling pathways.

S1P_4_ has emerged as a key pathogenic factor in several inflammatory diseases, including psoriasis [24], MASH [14], breast cancer [25], and transient ischemic stroke [13]. The involvement of S1P_4_ in these diseases underscores its critical role in inflammatory responses, making it a promising target for therapeutic interventions in conditions driven by chronic inflammation and immune dysregulation. Moreover, a recently developed functional antagonist of S1P_4_, NXC736 [13], has demonstrated remarkable therapeutic effects against transient ischemic stroke [13], MASH [14], and lung fibrosis [15]. In our previous study, we showed that blocking S1P_4_ activity, either by using a pharmacological antagonist (NXC736) or via genetic deletion (AAV-based S1P_4_ knockdown), attenuated transient ischemic stroke-induced brain damage during both acute and chronic phases [13]. In particular, the neuroprotective effects of NXC736 [13] indicate its potential as a promising therapeutic agent against transient ischemic stroke. Likewise, the current study also revealed the neuroprotective effects of NXC736 using a permanent ischemic stroke model, a clinically relevant model that simulates acute injuries observed in most clinical stroke cases [18]. NXC736 substantially alleviated brain damage in pMCAO-challenged mice during the acute phase (1 or 3 days) by attenuating the infarction volume, neurological functional deficits, and apoptotic cell death. Combined with previous findings in a mouse model of transient ischemic stroke [13], our results in permanent ischemic stroke mice indicate that NXC736 exerts neuroprotective effects against both transient and permanent ischemic stroke. Given that NXC736 is a functional antagonist of S1P_4_ [13,14], our findings suggest that S1P_4_ may contribute to the pathogenesis of brain injury in permanent ischemic stroke.

BBB disruption is a key pathological feature of ischemic stroke, characterized by increased permeability due to tight junction degradation and enhanced endothelial vesicle transport [26,27]. Loss of BBB tight junction integrity can enhance paracellular permeability, resulting in vasogenic edema, hemorrhagic transformation, and increased mortality [28]. This disruption is driven by ischemia-induced inflammation and immune activation [27]. In transient ischemic stroke, NXC736 intervention reduced neutrophil infiltration and improved the vasculature [13]. Similarly, in the current study, NXC736 enhanced the expression of tight junction proteins and improved BBB integrity in a permanent ischemia model, supporting its putative role in mitigating BBB dysfunction. Disrupted BBB also leaks serum proteins and intracellular substances into the extracellular space, leading to edema fluid accumulation and exacerbated ischemic brain edema [29] in permanent ischemic stroke. Additionally, NXC736 remarkably reduced brain water content and edema in the pMCAO-challenged brain, highlighting the potential effects of NXC736 in alleviating BBB disruption and vasogenic edema in permanent ischemia.

In the injured brains of ischemic stroke, neuroinflammatory responses, such as microglial activation and pro-inflammatory cytokine production, are well-known core pathogenic events [30]. Microglial activation is responsible for generating several factors that contribute to ischemic injury through neuroinflammation [31]. It is more prominent in brains injured by a stroke challenge, as evidenced by increased numbers of activated microglia, proliferation, and morphological conversion of ramified cells into amoeboid cells [32]. These events have also been observed in the pMCAO model [33]. In the current study, NXC736 markedly attenuated the increase in the number of activated microglia, proliferation, and morphological conversion of cells in pMCAO-challenged brains, suggesting that the NXC736-mediated neuroprotective effects in permanent ischemic stroke are associated with microglial activation. Activated microglia contribute to neuronal injury by producing pro-inflammatory cytokines (TNF-α, IL-23, IL-1β, IL-12, and IL-6), but they also promote repair through the production of anti-inflammatory cytokines (TGF-β1, IL-10, and IL-4) [34,35]. Levels of cytokines and chemokines, well-recognized mediators of the inflammatory process, are elevated after a stroke challenge. Moreover, anti-inflammatory compounds and abolition of pro-inflammatory genes are neuroprotective in ischemic stroke [36,37]. In the current study, NXC736 substantially attenuated the upregulation of pro-inflammatory cytokines (TNF-α, IL-1β, and IL-6) in the pMCAO-challenged brains, suggesting that the NXC736-mediated neuroprotective effects are associated with the modulation of pro-inflammatory cytokine production in permanent ischemic stroke.

Oxidative stress represents another critical pathological process in ischemic stroke [38]. Excessive production of reactive oxygen species (ROS) during ischemic insult leads to lipid peroxidation, protein oxidation, and mitochondrial dysfunction, which collectively exacerbate neuronal death and neuroinflammatory responses [39,40]. Among various ROS-generating enzymes, NOX4 has been identified as a major source of ROS contributing to ischemia-induced oxidative stress and neuronal injury [41]. In the present study, NXC736 markedly reduced both 4-HNE and NOX4 expression in the ischemic brain one day after pMCAO, indicating that NXC736 effectively suppresses oxidative stress and its downstream effects. This suppression is consistent with the overall neuroprotective effects of NXC736 on microglial activation, cytokine production, and BBB dysfunction. Therefore, attenuation of oxidative stress may contribute to the neuroprotective effects of NXC736 in permanent ischemic stroke.

In ischemic stroke, oxidative stress can amplify ischemia-induced inflammatory responses by triggering the release of pro-inflammatory cytokines and activating downstream signaling pathways, including NF-κB and MAPKs (ERK1/2, JNK, and p38) [42,43,44]. NF-κB activation, particularly in microglia, promotes the expression of cytokines such as TNF-α and IL-1β, amplifying neuroinflammation [45]. Similarly, MAPK signaling in glial and neuronal cells contributes to the production of inflammatory mediators [46]. The NF-κB and MAPK pathways act synergistically to establish a feed-forward loop that amplifies the inflammatory cascade and aggravates neuronal damage in ischemia [23]. Notably, the pathogenic function of S1P_4_ in stroke is closely associated with the activation of NF-κB and MAPKs (ERK1/2, JNK, and p38), which has recently been demonstrated in transient ischemic stroke [13]. In this study, NXC736 reduced NF-κB expression in microglia and attenuated the activation of MAPKs (ERK1/2, JNK, and p38) in the pMCAO-challenged brain, indicating that NXC736 may exert its protective effects in permanent ischemic stroke by modulating NF-κB and MAPK pathways.

In conclusion, our findings revealed that NXC736 markedly reduced brain damage in a permanent ischemic stroke model, along with its effects on relevant pathogenesis. Together with our previous findings in a transient ischemic stroke model, the current results obtained using a permanent ischemic model clearly demonstrate that NXC736 exerts neuroprotective effects against ischemic stroke. Because NXC736 can suppress the activity of S1P_4_, these findings further suggest that targeting S1P_4_ could be a promising therapeutic strategy for developing novel treatments for ischemic stroke.

## 4. Materials and Methods

### 4.1. Animals

Six-week-old male ICR mice (Orient Bio, Seongnam-si, Republic of Korea) were acclimatized for one week in a controlled environment before experimentation, with food and water provided *ad libitum*. The animal protocol for this study was approved by the Gachon Institutional Animal Care and Use Committee (GIACUC; GIACUC-R2021011). Animal handling and experiments were performed in strict accordance with the Animal Care and Use Guidelines provided by Gachon University (Incheon, Republic of Korea).

### 4.2. Permanent Ischemic Model and Drug Administration

Mice were subjected to pMCAO using the intraluminal suture method, as described previously [47]. Briefly, mice were deeply anesthetized with isoflurane, positioned supine under a stereo-dissecting microscope, and the ventral neck was sterilized before making a 1 cm midline incision. The right common carotid artery was separated and ligated, while the external carotid artery (ECA) was ligated. MCAO was induced by inserting a 5-0 monofilament coated with silicone (9 mm long with 0.21–0.22 mm tip diameter) from the point of bifurcation of the ECA and internal carotid artery (ICA) to the middle cerebral artery (MCA), followed by the ligation of the ICA. For sham-operated mice, the same surgical procedure was performed, except for MCA occlusion. During the entire procedure, the body temperature of the mouse was maintained at 37 °C using a heat lamp, and lidocaine was applied after the surgery for pain control. The pMCAO-challenged mice were randomly allocated to two treatment groups: vehicle (10% Tween 80 in distilled water) or NXC736 (3 mg/kg; selected based on optimal efficacy in our previous transient MCAO mouse study [13]). Both the vehicle and drug were administered orally (*p.o.*) 1 h post-occlusion.

### 4.3. Determination of Neurological Score

A modified neurological severity score was used to determine functional neurological deficits. As described in previous studies [13,47], the neurological deficit score comprises scores for motor, sensory, balance, and reflex tests on an 18-point scale (0 for normal and 18 for maximum deficits).

### 4.4. Determination of Brain Infarction Volume

The brain infarction volume was measured one or three days after pMCAO challenge, as described previously [47]. Briefly, the mice were sacrificed by CO_2_ inhalation, and their brains were harvested immediately. The brains were cut into 2 mm thick slices using a coronal brain matrix. Brain slices were stained with 2% 2,3,5-triphenyltetrazolium chloride in saline at 37 °C for 15 min. The stained slices were photographed, and the brain infarction volume was analyzed using the ImageJ software (Version 1.54g; National Institute of Mental Health, Bethesda, MD, USA).

### 4.5. Reverse Transcription-Quantitative PCR (RT-qPCR) Analysis

Total RNA was isolated from the damaged hemisphere of the brain tissue one or three days after pMCAO or sham challenge using RNAiso plus (Takara, Kusatsu, Japan). First, RNA was reverse-transcribed into cDNA using the All-in-One First-Strand cDNA Synthesis SuperMix (TransGen Biotech, Beijing, China). RT-qPCR was performed using a StepOnePlusTM qRT-PCR system (Applied Biosystems, Foster City, CA, USA) with Power SYBR Green PCR master mix (Life Technologies, Carlsbad, CA, USA). mRNA expression levels were quantified using the 2^−ΔΔCT^ method and normalized to the housekeeping gene β-actin. The primer sequences are listed in Table 1.

### 4.6. Western Blot Analysis

Proteins were extracted from the damaged hemisphere of each brain. Briefly, tissues were homogenized using a tissue homogenizer and protein lysis buffer containing a protease inhibitor cocktail and centrifuged at 4 °C. Prepared protein samples were separated by 10% SDS-PAGE and transferred to methanol-activated polyvinylidene difluoride (Merck Millipore, Burlington, MA, USA). The membranes were blocked with 5% skim milk for 1 h at room temperature and incubated overnight at 4 °C with respective primary antibodies against Bcl-2 (1:1000; Cell Signaling Technology, Beverly, MA, USA), cleaved caspase-3 (1:1000; Proteintech, Rosemont, IL, USA), 4-hydroxynonenal (4-HNE; 1:1000; Bioss, Freiburg, Germany), NADPH oxidase 4 (NOX4) (1:1000; Abcam, Cambridge, UK), NF-κB (1:200; Cell Signaling Technology), phosphorylated forms of MAPKs (p-ERK1/2, p-JNK, and p-p38; 1:1000; Cell Signaling Technology), total forms of MAPKs (ERK1/2, p38, and JNK; 1:1000; Cell Signaling Technology), and β-actin (1:10,000; Bethyl Laboratories, Montgomery, TX, USA). The following day, the membranes were washed three times with phosphate-buffered saline (PBS) containing 0.3% Triton X-100 (PBST) and incubated with horseradish peroxidase-conjugated secondary antibodies (1:10,000; Santa Cruz Biotechnology, Dallas, TX, USA). Protein bands were detected using an enhanced chemiluminescence detection kit (Dongin Biotech Co., Seoul, Republic of Korea). The expression levels of target protein bands were quantified using ImageJ and normalized to the respective β-actin.

### 4.7. Tissue Preparation for Histological Analysis

Brain samples were obtained one or three days after pMCAO for histological analyses. Briefly, mice were deeply anesthetized with isoflurane for transcardial perfusion with ice-cold PBS and fixed with 4% paraformaldehyde (PFA). The harvested brains were further post-fixed in 4% PFA overnight and immersed in 30% sucrose solution for dehydration the following day. Dehydrated brains were embedded in Tissue-Tek optimal cutting temperature compound, frozen on dry ice, and coronally sectioned (20 µm) using a cryostat (RD-2230; Roundfin, Shenyang, Liaoning, China).

### 4.8. FJB Staining

Brain cell death was assessed by FJB staining. Frozen sections were warmed in a slide warmer, rinsed with water, immersed in an alcohol series (100% ethanol for 3 min, 70% ethanol for 1 min, and 30% ethanol for 1 min), and washed with water for 1 min. The sections were oxidized in 0.06% potassium permanganate for 15 min, washed with water, stained with 0.001% FJB in 0.1% acetic acid solution for 30 min, and rinsed with water. Thereafter, the rinsed sections were dried in the dark, dehydrated with xylene, mounted with Entellan media (Merck, Darmstadt, Germany), and dried.

### 4.9. Iba1 and 4-HNE Immunohistochemistry

Microglial activation and lipid peroxidation were assessed by immunohistochemical staining for Iba1 and 4-HNE, respectively. Brain sections were treated with 1% hydrogen peroxide (H_2_O_2_) for oxidation, blocked with 1% fetal bovine serum (FBS), and incubated with rabbit anti-Iba1 (1:500; Wako Pure Chemicals, Osaka, Japan) or rabbit anti-4-HNE (1:750; Bioss) overnight at 4 °C. Subsequently, sections were incubated with a biotinylated secondary antibody (1:200; Santa Cruz Biotechnology) for 2 h at room temperature and further incubated with ABC reagent (1:100; Vector Laboratories, Newark, CA, USA). Thereafter, sections were developed using a 3,3′-diaminobenzidine kit (Dako, Santa Clara, CA, USA), washed in distilled water, dehydrated with alcohol and xylene, and mounted with Entellan mounting medium (Merck, Darmstadt, Germany).

### 4.10. BrdU/Iba1 Double Immunofluorescence

Microglial proliferation was determined by BrdU/Iba1double immunofluorescence. Briefly, mice were administered 50 mg/kg BrdU (Sigma-Aldrich, St. Louis, MO, USA) intraperitoneally every 12 h for 2 days. The prepared brain sections were post-fixed in 4% PFA, DNA denatured with 2N HCl, neutralized with 0.1 M borate buffer (pH 8.5), blocked for 1 h at 4 °C with 1% FBS in PBST, and then incubated overnight at 4 °C with primary antibodies: rabbit anti-Iba1(1:500), and rat anti-BrdU (1:200; Abcam). The following day, the sections were labeled with secondary antibodies conjugated to Cy3 (1:1000; Jackson ImmunoResearch, West Grove, PA, USA) and Alexa-Fluor^®^488 (AF488; 1:1000; Jackson ImmunoResearch) and mounted with VECTASHIELD (Vector Laboratories, Newark, CA, USA).

### 4.11. NF-κB/Iba1 Double Immunofluorescence

NF-κB/Iba1 double immunofluorescence analysis was performed to assess NF-κB expression in the microglia. Sections were post-fixed with 4% PFA and blocked for 1 h at 4 °C with 1% FBS in PBST. Subsequently, sections were incubated overnight at 4 °C with primary antibodies against mouse anti-NF-κB (1:200; Santa Cruz Biotechnology) and rabbit anti-Iba1 (1:500). The next day, the sections were labeled with secondary antibodies conjugated to Cy3 (1:1000) and AF488 (1:1000) and mounted with VECTASHIELD.

### 4.12. Claudin-5/CD31 Double Immunofluorescence

Claudin-5 expression in endothelial cells was assessed by Claudin-5/CD31 double immunofluorescence staining. Sections were post-fixed with 4% PFA, blocked for 1 h at 4 °C with 1% FBS in PBST, and incubated overnight at 4 °C with anti-Claudin-5 (1:300; Santa Cruz Biotechnology) and anti-CD31 (1:300; Dianova, Hamburg, Germany) antibodies. The following day, sections were labeled with AF488- or Cy3-conjugated secondary antibodies.

### 4.13. Calculation of Brain Edema

Brain water content and swelling were determined to analyze brain edema. We measured the water content in the brain tissue of the mice using the dry–wet method. The wet weight of the brain was measured immediately after decapitation. For dry weight, brain tissue was wrapped with tin foil and maintained at 100 °C for 24 h. The percentage of brain water content was calculated using the following formula: Brain water content (%) = (wet weight − dry weight)/wet weight. To calculate the extent of swelling, the Kaplan method was used: extent of edema = (the volume of right hemisphere − the volume of left hemisphere)/the volume of left hemisphere.

### 4.14. Image Preparation and Quantification

After immunohistochemistry, brain sections were imaged using a bright-field or fluorescence microscope equipped with a DP72 camera (BX53T; Olympus Co., Tokyo, Japan). Representative images were processed using Adobe Photoshop Elements 8. To quantify immunopositive cells, four images from each brain region were used, and the mean value was used for the number of cells in each brain region. The number of cells was represented as the number of cells per unit area.

### 4.15. Statistical Analysis

Data were analyzed using GraphPad Prism 8 (Version 8.2.1; GraphPad Software Inc., La Jolla, CA, USA) and are presented as mean ± S.E.M. Data distribution was evaluated using the Shapiro–Wilk test. When data followed a normal distribution, statistical significance was determined using a one-way ANOVA followed by Tukey’s post hoc test for multiple comparisons or a Student’s *t*-test for pairwise comparisons. For non-normally distributed data, the Kruskal–Wallis test followed by Dunn’s post hoc test was used for multiple group comparisons, and the Mann–Whitney test was applied for two-group comparisons. Statistical significance was defined as *p* < 0.05.

## Figures and Tables

**Table 1:** Primer sequences used for RT-qPCR.

Target	Forward	Reverse
β-actin	5′-AGCCTTCCTTCTTGGGTATG-3′	5′-CTTCTGCATCCTGTCAGCAA-3′
TNF-α	5′CATCTTCTCAAAATTCGAGTGACAA-3′	5′-TGGGAGTAGACAAGGTACAACCC-3′
IL-1β	5′-CAACCAACAAGTGATATTCTCCATG-3′	5′-GATCCACACTCTCCAGCTGCA-3′
IL-6	5′-GAGGATACCACTCCCAACAGACC-3′	5′-AAGTGCATCATCGTTGTTCATACA-3′
TGF-β1	5′-CAACCCAGGTCCTTCCTAAA-3′	5′-GGAGAGCCCTGGATACCAAC-3′
IL-4	5′-GTCATCCTGCTCTTCTTTCTCG-3′	5′-TCTGTGGTGTTCTTCGTTGCT-3′
IL-10	5′-TGGCCTTGTAGACACCTTGG-3′	5′-AGCTGAAGACCCTCAGGATG-3′

## Data Availability

The data used to support the findings of this study are available from the corresponding authors upon request.

## Abbreviations

The following abbreviations are used in this manuscript:

AAV	Adeno-associated virus
BBB	Blood–brain barrier
BrdU	Bromodeoxyuridine
CCA	Common carotid artery
ECA	External carotid artery
ERK1/2	Extracellular signal-regulated kinase 1/2
FTY720	Fingolimod
FJB	Fluoro-Jade B
ICA	Internal carotid artery
IL	Interleukin
Iba1	Ionized calcium-binding adapter molecule 1
JNK	c-Jun N-terminal kinase
MAPKs	Mitogen-activated protein kinases
MASH	Metabolic dysfunction-associated steatohepatitis
MCAO	Middle cerebral artery occlusion
NF-κB	Nuclear factor kappa-light-chain-enhancer of activated B cells
pMCAO	permanent middle cerebral artery occlusion
S1P_4_	Sphingosine-1-phosphate receptor 4
TGF-β1	Transforming growth factor-β1
TNF-α	Tumor necrosis factor-α
